# Changes in peripheral blood lymphocytes in polycythemia vera and essential thrombocythemia patients treated with pegylated-interferon alpha and correlation with *JAK2*^V617F^ allelic burden

**DOI:** 10.1186/s40164-016-0057-y

**Published:** 2016-09-27

**Authors:** Magdalena Kovacsovics-Bankowski, Todd W. Kelley, Olga Efimova, Soo Jin Kim, Andrew Wilson, Sabina Swierczek, Josef Prchal

**Affiliations:** 1 Division of Hematology and Hematological Malignancies, School of Medicine, University of Utah School of Medicine, Salt Lake City, UT 84132 USA; 2 Department of Pathology and ARUP Institute for Clinical and Experimental Pathology, University of Utah School of Medicine, Salt Lake City, 84108 UT USA; 3 School of Nursing, University of Utah School of Medicine, Salt Lake City, 84112 UT USA

**Keywords:** Myeloproliferative neoplasms, Pegylated-interferon alpha, Regulatory T cells, Immune monitoring, Immune checkpoint, *JAK2*^V617F^

## Abstract

**Background:**

Pegylated-interferon alpha (PegINFα) treatment of patients with polycythemia vera (PV) and essential thrombocythemia (ET) has resulted in long-term clinical response, decreased *JAK2* ^V617F^ allelic burden and restoration of polyclonal hematopoiesis. The mechanisms of the beneficial effects of PegINFα are not clear, but available evidence suggests direct suppression of *JAK2*-mutated clone, induction of dormant stem cells to proliferation, and augmentation of an immune effect against PV and ET clones.

**Methods:**

We analyzed the phenotype and frequency of peripheral blood lymphocytes (PBL) from PegINFα treated patients and compared them to patients treated with hydroxyurea (HU). Samples collected at various time points before and during treatment were analyzed using multicolor flow cytometry.

**Results:**

We found that PegINFα increased the frequency of peripheral blood CD4^+^ Foxp3^+^ regulatory T cells (Treg). Highly suppressive Treg, characterized by co-expression of CD39 and HLA-DR, were also increased in PBL from PegINFα treated patients. We observed an augmentation of cycling CD8^+^ T cells, NK cells, and of poorly activated CD38^+^CD8^+^ T cells. Our results also suggest that PegINFα increased the frequency of PD-1^+^ CD4^+^ helper cells and PD-1^+^ CD4^+^ Foxp3^+^ Treg cells. None of these changes were present in HU treated patients. We analyzed the correlation between changes in different T cell populations in the peripheral blood with the changes in *JAK2* ^V617F^ allelic burden in clonal granulocytes. Augmentation of Ki-67^+^ Treg, HLA-DR^+^ CD39^+^ Treg, Helios^+^ Treg and HLA-DR^+^ CD38^+^ CD8^+^ T cells correlated with an increase in *JAK2* ^V617F^ allelic burden. We also found a positive correlation between PD-1^+^ Treg and *JAK2* ^V617F^ allelic burden; however, the number of available patients was small (n = 7).

**Conclusions:**

We report marked changes in frequencies of PBL subsets after PegINFα treatment, suggesting an immunomodulatory effect by PegINFα. Generation of a more suppressive immune response, as measured by an increase in highly suppressive Treg and poorly activated CD8^+^ T cells, correlated with a poor molecular response. In this study, we have not identified changes in the PBL that would indicate the presence of an effective anti-tumor response.*Trial registration* NCT01259856, December 7. 2010 and NCT01259817, December 6. 2010, Grant #1P01CA108671-O1A2, July 17. 2006, Sponsor: MPDRC/NIH, NCI-2012-00269, January 12. 2011 and NCI-2012-00268, January 12. 2011

**Electronic supplementary material:**

The online version of this article (doi:10.1186/s40164-016-0057-y) contains supplementary material, which is available to authorized users.

## Background

Polycythemia vera (PV) and essential thrombocytosis (ET) are myeloproliferative neoplasms (MPN) characterized by the excessive proliferation but unhindered differentiation of a hematopoietic stem cell clone. The *JAK2* ^V617F^ mutation is present in more than 95 % of patients with PV and more than 50 % of patients with ET [1–4]. Chemotherapy decreases cell counts and thrombotic events by suppressing proliferation of PV and ET clones, but only interferon alpha (INFα) has occasionally restored polyclonal hematopoiesis [5] and often reduced *JAK2* ^V617F^ allelic burden [6]. However, there are multiple mechanisms by which INFα promotes this response, including a direct suppression of *JAK2* mutated hematopoietic progenitors [7, 8], stimulation of quiescent residual normal cells by promoting cell division, differentiation of hematopoietic stem and progenitor cells [9], or beneficial alteration of humoral and cellular immune responses.

The role of T cells in tumor immunity is well established. The recent successes of immunomodulatory therapies such as antibodies targeting checkpoint molecules or adaptive T cell therapy have clearly established the role of immunotherapy in the treatment of a variety of cancers, such as melanoma, renal carcinoma and non-small lung cancer [10, 11]. It was previously reported that INFα increases function of CD4 effector T-cells, CD8^+^ T-cells and B-cells and that it may exert anti-tumor effects. [12–14]. INFα effects on Treg are not fully understood. However, some studies have shown an activating effect of INFα on Treg [15, 16]. Thus, in melanoma patients, INFα treatment is used as an adjuvant therapy [17] and the systemic immune effects of INFα in these patients have been described [18].

Failure of the immune system to control clonal growth is very often due to immunological imbalance. In many cancers, Treg are increased and contribute to the failure of the immune system to clear the tumor. Several types of Treg with distinct functional differences have been recognized. Treg that co-express CD39 and HLA-DR are more suppressive than Treg that do not express these two surface markers [19]. Another highly suppressive Treg subset is characterized by expression of the Ikaros family member transcription factor Helios [20]. A decrease in a highly suppressive CD39^+^ and HLA-DR^+^ Treg subset has been correlated with a decreased tolerance, resulting in kidney graft rejection or pre-term pregnancy loss [21, 22]. Increased Helios^+^ Treg have been observed in tumor infiltrating lymphocytes [23].

To be fully functional, CD8^+^ T cells need to be stimulated along a well-defined pathway [24]. First, a T cell receptor is engaged by its cognate MHC-peptide complex. Then a second signal is delivered when CD28 expressed at the surface of activated T cells is engaged by B7 present at the surface of antigen presenting cells. To fully activate T cells, a third signal is necessary and is provided by the engagement of OX40 by its ligand OX40 ligand. OX40 is a member of the tumor necrosis superfamily expressed at the surface of recently activated T cells [25]. Failure to accomplish these three steps leads to poorly activated, hypofunctional T cells characterized by co-expression of CD38 and HLA-DR. Non-specific activation, or bystander activation, is often observed in cancer or acquired immunodeficiency syndrome [26]. In patients infected with human immunodeficiency virus, the presence of CD38^+^ HLA-DR^+^ CD8^+^ T cells correlates with early progression of disease [27].

To prevent overactivation of the immune system, several checkpoint molecules control T cell activation and prevent accumulation of activated T cells. CTLA-4, a member of the immunoglobulin superfamily, upon binding of its ligand (CD80 or CD86), inhibits proliferation of effector T cells. PD-1, a member of the CD28 family, is expressed on T cells upon activation, and its engagement by PDL-1 or PDL-2, results in T cell death, anergy and exhaustion. High levels of CTLA-4 or PD-1 expression on tumor infiltrating T cells contributes to dysregulation of the immune response in cancer patients.

In this study, we attempted to determine whether PegINFα stimulation of the immune system contributes to the beneficial response to PegINFα therapy. PegINFα is a form of INFα with a longer half-life, less toxicity and more convenient administration. Riley and colleagues have reported an increase in circulating NK cells and Treg in MPN patients treated with PegINFα [28–30]; however, no correlation with clinical, cytological, or molecular response to therapy was reported. This study confirms increased circulating NK cells and Treg after PegINFα therapy and provides a more detailed characterization of PBL in patients treated with PegINFα. We report a positive correlation between changes in *JAK2* ^V617F^ allelic burden and changes in lymphocyte populations in the peripheral blood. These changes are independent of myelosuppression, as they were not observed in PV and ET patients treated with HU. We hypothesize that the increase in CD39^+^ HLA-DR^+^ Treg contributes to suppression of the immune response, resulting in treatment failure. We suggest that increased CD39^+^ HLA-DR^+^ Treg could be used as a biomarker to predict if Peginfα treatment will have beneficial effects in MPN patients.

## Methods

### Study subjects

This study included the following three groups of subjects: (1) patients who participated in MPD-RC 112, a Phase 3 randomized trial in high-risk PV and ET using PegINFα versus HU; (2) patients who participated in MPD-RC 111 for PV and ET patients intolerant or refractory to hydroxyurea and patients with Budd Chiari syndrome (both trials are part of the Myeloproliferative Disorders Research Consortium); and (3) patients who did not participate in these protocols. Peripheral blood samples were collected prior to and at different time points during treatment, after patients signed an approved Institutional Review Board informed consent. Patient’s characteristics are described in Table 1.

**Table 1:** Patient’s characteristic

	PegIFNα (n = 25)	Hydroxyurea (n = 7)
Age	57.0	64
Median (range)	(39–86)	(61–77)
*Gender*		
Male/female	14/12	5/2
*Diagnosis*		
PV/ET/ET-MF	18/7/1	5/1/1
*JAK2* ^V617F^ allelic burden	25.4	60
Median (range)	(1.0–99.2)	(4.54–93.3)
Hematocrit %	46.9	50.5
Median (range)	(28.7–70)	(33.4–70.2)
WBC ×10^9^/L	8.1	8.0
Median (range)	(2.5–17.3)	(4.3–12.5)
Platelets × 10^9^/L	391	393
Median (range)	(112–1555)	(123–878)

### Trial registration

NCT01259856, December 7. 2010, NCT01259817, December 6. 2010, IRB_00017793, grant #1P01CA108671-O1A2, July 17. 2006, Sponsor: MPDRC/NIH, IRB_ 45342, NCI-2012-00269, January 12. 2011 and IRB_36791, NCI-2012-00268, January 12. 2011.

### Blood sample processing

10 mL of peripheral blood were obtained by venipuncture. Granulocyte and mononuclear cell fractions were isolated according to previously published protocols [31, 32]. After isolation, PBMC were frozen and stored prior to analysis.

### Flow cytometry

Single cell suspensions of leukocytes from peripheral blood were stained using the following monoclonal antibodies: anti-CD3 APC-H7 (BD, 641397), anti-CD4 Alexa Fluor 700 (BD, 557922), anti-CD8 Alexa Fluor 700 (BD, 557945), anti-CD25 APC (Miltenyi 130-092-858), anti-CD28 BV421(BD, 562613), anti-CD38 BV605 (Biolegend 303532), anti-HLA-DR PE-Cy7 (BD Biosciences, 335795), anti-CD39 BV421 (BD 563679), anti-CD45 RO BV510 (BD, 563215), anti-CD56 APC (Biolegend 318310), anti-CCR7 PE (BD 561008), anti-FoxP3 PE (eBioscience, 12-4777-42), anti-Ki-67 FITC (BD 556026), anti-HELIOS (Biolegend, 137214), anti-CTLA-4 APC (BD 560938), anti-OX40 FITC (Biolegend 35006), and anti-PD1 BV510 (Biolegend 329932). Cells were stained in FACs buffer (1 % FBS in PBS with 0.01 % NaN_3_) and fixed according to the eBioscience FoxP3 Fix-Perm kit protocol (eBioscience, 00-5521-00). All samples were analyzed on a BD X20 Fortessa Flow cytometer. We used Fluorescence Minus One to discriminate between positive and negative cells for each antibody [33].

### DNA isolation and *JAK2*^V617F^ analysis

Genomic DNA was isolated from granulocytes using Puregene DNA purification kits (Gentra, MN, USA). The *JAK2* ^V617F^ mutational burden was determined by quantitative real-time allele specific PCR (qRT-PCR) employing a sequence 7500 detection system (Applied Biosystems, Foster City CA, USA) in which an allele-specific primers, containing both a mismatched nucleotide and a locked nucleic acid, are used to enhance discrimination between polymorphic nucleotides, as previously described [34].

### PBL and *JAK2*^V617F^ correlation analysis

Correlation matrices were created to identify potential bivariate linear relationships between changes in peripheral blood Treg populations and changes in *JAK2* ^V617^ allelic burden. Significantly correlated variables, with corresponding Pearson’s product-moment correlation coefficient (r) and p value, are presented in Fig. 6. A generalized linear model (GLM) was calculated for each pair as well. However, for interpretation, r was preferable to the beta coefficients to include in Fig. 6. Analysis was performed adapting the cor.test and corrplot functions using R software version 3.2.3 (Copyright (C) 2015 The R Foundation for Statistical Computing).

## Results

### PegINFα increases the frequency of CD4^+^ Foxp3^+^ Treg and highly suppressive Treg in peripheral blood

Under the approval of our institute’s Institutional Review Board, 32 patients with MPN were enrolled in the study. As described in Table 1, 25 patients were treated with PegINFα and 7 with HU.

**Fig. 1:**
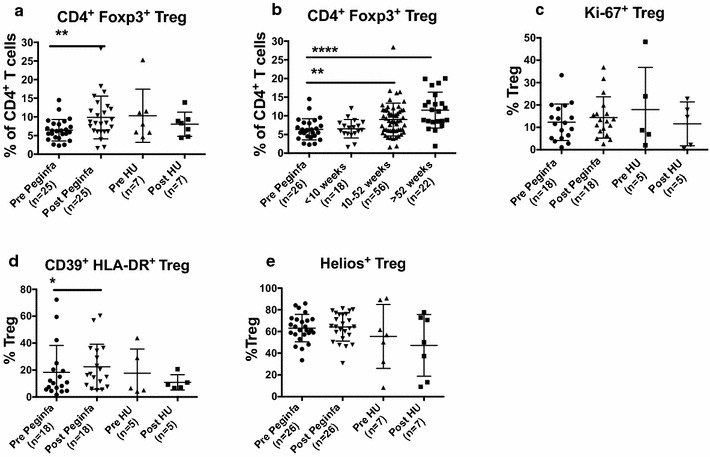
PegINFα increases Treg and highly suppressive Treg cells in peripheral blood of PV and ET patients. PBMC were collected from patients with PV or ET treated with PegINFα or HU for at least 70 days (range 70–616, median 112 for PegINFα and range 113–2422, median 175 for HU treated patients). Lymphocytes were analyzed by flow cytometry using surface markers, CD3, CD4, CD25, CD39, HLA-DR and intracellular markers Foxp3, Ki-67 and Helios. **a** represents CD4^+^ CD25^+^ Foxp3^+^ Treg cells. In **b** the frequency of Treg was analyzed at different time points after initiation of PegINFα treatment. **c–e** show frequency of Ki-67^+^ Treg, Helios^+^ Treg, and CD39^+^/HLA-DR^+^) Treg

Peripheral blood samples were received and processed as described in “Methods” section. To determine the effects of PegINFα on peripheral blood Treg, we performed multicolor flow cytometry analysis with antibodies that recognize CD3, CD4, CD25, Foxp3, CD39, HLA-DR, Helios and Ki-67. Changes in T-cell subpopulations were analyzed by prism using fluorescence minus one as internal controls. Peripheral blood mononuclear cells (PBMC) were gated on CD3 and CD4 and then further analyzed for Ki-67, a marker of cell proliferation, as well as Foxp3 and CD25, markers of Treg. Treg were further analyzed for co-expression of CD39 and HLA-DR, and expression of Helios. Treg expressing these markers represent Treg that are more suppressive. An example of flow cytometry analysis is shown in Additional file 1: Fig. S1. As shown in Fig. 1a, CD4^+^ Foxp3^+^ Treg increased from 6.4 to 9.9 %, p = 0.0017, in patients receiving PegINFα, while no change was observed in HU treated patients. This increase continued with time and was higher after 52 weeks of treatment at 11.4 % (Fig. 1b).

For 18 of 25 patients treated with PegINFα and 5 of 7 HU treated patients, we were able to perform a more detailed analysis of the Treg population. The proliferation of Treg as measured by Ki-67 was not significantly increased in PegINFα treated patients (12.3–14.4 % p = 0.5509) (Fig. 1c). When analyzing the more suppressive phenotype of Treg, we observed that PegINFα significantly increased the proportion of CD39^+^ HLA-DR^+^ Treg from 10.6 to 16.7 %, p = 0.0139 (Fig. 1d). No significant increase in the proportion of Helios^+^ Treg (63.15–64.2 % p = 0.2188) was observed in PegINFα treated patients (Fig. 1e). There was no statistically significant change in the absolute number of CD39^+^ HLA-DR^+^, Ki-67^+^ or Helios^+^ Treg in Peginfa treated patients (Additional file 2: Fig. S2). We did not observe any significant changes in Treg subpopulations in HU treated patients.

### Proliferation and phenotypic changes in CD8^+^ T cells induced by PegINFα

**Fig. 2:**
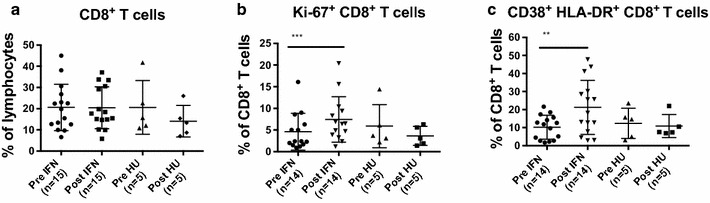
Increased proliferation and activation of CD8^+^ T cells in peripheral blood of PegINFα treated patients. PBMC collected prior to and after at least 10 weeks of treatment were analyzed using anti-CD3, -CD8, -CD38, -HLA-DR and Ki-67 antibodies. Percentage of CD8^+^ T cells, cycling (Ki-67^+^) CD8^+^ T cells and CD38^+^/HLA-DR^+^ CD8^+^ T cells are shown in (**a**–**c**) respectively

Next, we analyzed CD8^+^ CD3^+^ cytotoxic T cells for expression of CD28, Ki-67, CD38 and HLA-DR before and after therapy with PegINFα or HU. The frequency of CD8^+^ T cells was not affected by PegINFα (20.3 and 20.1 %) or HU (21.8 and 13.8 %), respectively (Fig. 2a). However, in PegINFα treated patients, a higher proportion of CD8^+^ T cells co-expressed Ki-67, indicating increased proliferation (4.6–7.5 %, p = 0.001). HU did not induce proliferation of CD8^+^ T cells (Fig. 2b). Moreover, we observed phenotypic differences in CD8^+^ T cells, including a significant increase in the CD38^+^ HLA-DR^+^ CD8^+^ subset, representing activated cells, in patients receiving PegINFα (10.2 % before treatment and 21.2 % during treatment, p = 0.0015; Fig. 2c). No changes were observed in HU treated patients.

### PegINFα induces expression of checkpoint molecules on T cells

**Fig. 3:**
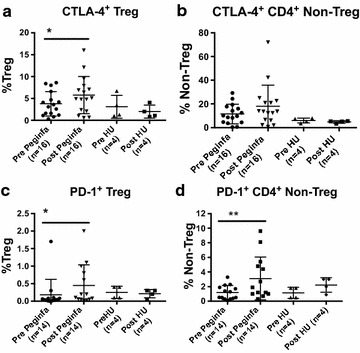
Expression of checkpoint molecules is increased by PegINFα. CD3^+^ CD4^+^T cells in peripheral blood were separated into Treg and non-Treg based on the expression of Foxp3. Foxp3^+^ Treg, (**a** and **c**) or Foxp3^−^ non-Treg (**b** and **d**) were examined for expression of CTLA-4 (**a**–**b**) and PD-1 (**c**–**d**)

We investigated the expression of checkpoint molecules PD-1 and CTLA-4 on CD3^+^ T cells in peripheral blood in 14 of 25 PegINFα treated patients and 4 of 7 HU treated patients. The frequency of peripheral blood CD4^+^ Treg and CD4^+^ effector T cells (Teff) expressing PD-1 increased in PegINFα-treated patients (0.18 % before treatment and 0.44 % during treatment for Treg, p = 0.016; and 1.2–3.1 % Teff, p = 0.0029) (Fig. 3c, d). The proportion of CTLA-4 expressing Treg was also augmented in patients treated with PegINFα (Fig. 3a, b). HU did not affect the expression of either PD-1 or CTLA-4.

### Increased CD3^−^ CD56^+^ NK cell proliferation

**Fig. 4:**
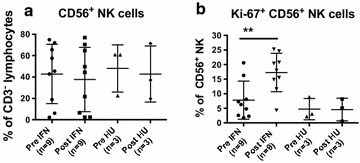
Increased CD3^−^/CD56^+^ NK cells proliferation in peripheral blood of PV and ET patients treated with PegINFα. PBMC were collected from patients prior to and after at least 10 weeks of treatment and analyzed for surface markers CD3 and CD56 and intra-cellular marker Ki-67. **a** shows CD56^+^ frequency and **b** shows frequency of Ki-67^+^ CD56^+^ NK cells

Analysis of CD3^−^ CD56^+^ NK cells was performed in 9 of 25 patients receiving PegINFα and 3 of 7 patients treated with HU. PegINFα did not affect the proportion of NK cells in the peripheral blood. However, the proportion of dividing NK cells was significantly increased, as shown in Fig. 4a, b. Prior to PegINFα treatment, 7.8 % of NK cells were proliferating; after at least 10 weeks of treatment, the frequency increased to 17.3 %, p = 0.0078. No changes were observed in HU treated patients.

### Correlation between peripheral blood T cells and molecular response in PegINFα treated patients

In 18 PV and 7 ET patients treated with PegINFα, molecular response was evaluated by measuring the *JAK2* ^V617F^ allelic burden. One ET patient was *JAK2* ^V617F^ negative and carried a calreticulin mutation and another ET patient was triple negative [35]. Patients were subdivided into two groups based on percentage reduction of *JAK2* ^V617F^ allelic burden. Patients with a 20 % or greater decrease in *JAK2* ^V617F^ allelic burden were categorized as responders (R), and those with a less than 20 % decrease were categorized as non-responders (NR). In this cohort, there were 10 R (9 PV and 1 ET) and 13 NR (9 PV and 4 ET).

**Fig. 5:**
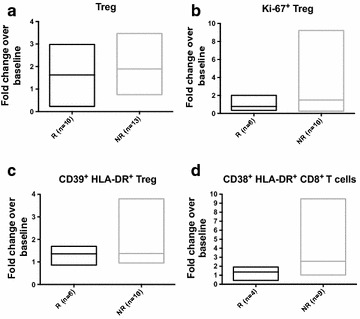
Changes in T cell subsets vary based on molecular response. Patients treated with PegINFα were separated into R and NR subsets based on a 20 % decrease in *JAK2* ^V617^ allelic burden. PBMC were analyzed as described above and fold changes compare to baseline were examined. **a** represents 23 patient samples (10R and 13 NR), **b**, **c** include 16 patients (6R and 10NR), and **d** includes 13 patients (4R and 9 NR). **a**–**c** show frequency of Treg, dividing Treg, CD39^+^ HLA-DR^+^ Treg and Helios^+^ Treg. **d** shows CD38^+^ HLA-DR^+^ T cells in R vs NR

We sought to determine whether changes in PBL correlated with changes in *JAK2* ^V617F^ allelic burden. As shown in Fig. 5, we observed several trends consistent with previously published data in other malignancies. NR showed a higher increase of Treg frequency, Treg proliferation and highly suppressive CD39^+^ HLA-DR^+^ Treg frequency. When looking at changes in absolute Treg, a similar trend was observed (Additional file 3: Fig. S3). A more pronounced increase of CD38^+^ HLA-DR^+^ CD8^+^ T cells were observed in NR.

### Changes in PBL correlate with *JAK2*^V617F^ allelic burden

**Fig. 6:**
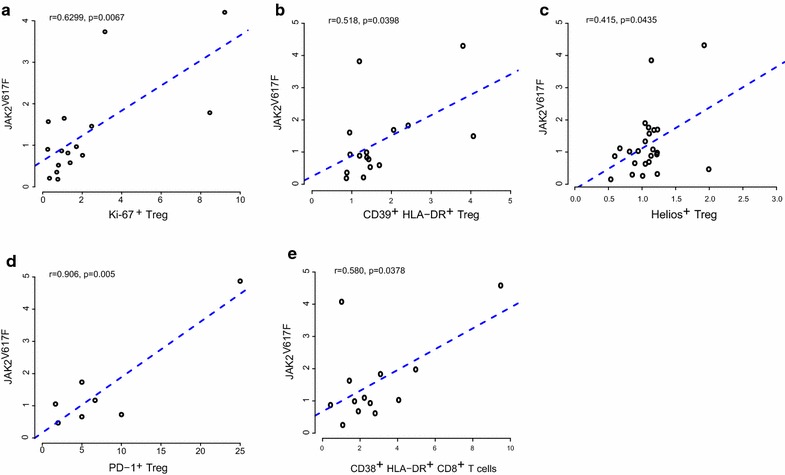
Correlation between increasing *JAK2* ^V617^ allelic burden in patients treated with PegINFα and augmentation of peripheral blood Treg populations. Changes in peripheral blood Treg population were correlated with changes in *JAK2* ^V617^ allelic burden. **a** Ki-67^+^ Treg, CD39^+^/HLA-DR^+^ Treg (**b**), Helios^+^ Treg (**c**), PD-1^+^ Treg (**d**) and CD38^+^ HLA-DR^+^ CD8^+^ T cells (**e**)

Using Spearman correlation analysis, we identified several Treg populations that increased in the peripheral blood and correlated with an increase in *JAK2* ^V617F^ allelic burden. An increased proportion of Ki-67^+^ Treg, HLA-DR^+^ CD39^+^ Treg and Helios^+^ Treg correlated with an increase in *JAK2* ^V617F^ allelic burden (Fig. 6a–c). Analysis of seven patients shows a strong positive correlation between PD-1^+^ Treg and *JAK2* ^V617F^ allelic burden (Fig. 6d). These data need to be confirmed in a larger number of patients. We observed that an increase in the CD38^+^ HLA-DR^+^ CD8^+^ T cell subset also correlated with an increases in the *JAK2* ^V617F^ allelic burden (Fig. 6e).

## Discussion

PegINFα is the only treatment for PV and ET patients reported to result in long-term clinical and molecular remission [34]. In some patients, we observed a marked decrease of *JAK2* ^V617F^ allelic burden that persisted even after discontinuation of treatment, suggesting a PegINFα-induced immune-mediated mechanism. INFα increases tumor immunogenicity, activates antigen presenting cells, and stimulates both CD4^+^ and CD8^+^ T cells [36, 37]. Several other studies analyzed PBL, characterized the effect of PegINFα and reported similar results [28, 29]. Previously, our group also reported an increase of Treg in PV and ET patients during therapy with PegINFα [38]. However, a detailed analysis of PBL subsets and their correlation with clinical and molecular response had not previously been established.

In this study, we used multi-color flow cytometry to analyze PBL of PV and ET patients treated with PegINFα or HU. We found an increase in peripheral blood Treg in patients treated with PegINFα. In addition, the frequency of highly suppressive CD39^+^ HLA-DR^+^ Treg was also increased. In the NR subset of patients, we found a trend towards increased frequency of Treg, increased proliferation of Treg and increased CD39^+^ HLA-DR^+^ Treg compared to the R group (p = 0.55, p = 0.2530, p = 0.4078 respectively). When looking at the absolute numbers of Treg, no significant changes were observed. However, since PegINFα decreased absolute lymphocyte counts, it was interesting to observe that CD39^+^ HLA-DR^+^ Treg numbers did not decrease.

We assessed if changes in PBL subpopulations predicted molecular response. We found a positive correlation between increased proliferating Treg (Ki-67^+^ Treg), highly suppressive Treg (HLA-DR^+^/CD39^+^ or Helios^+^ Treg) and *JAK2* ^V617F^ allelic burden. We also found a positive correlation between increased HLA-DR^+^ CD38^+^ CD8^+^ T cells and increased *JAK2* ^V617F^ allelic burden. Similar results were observed in hepatitis C patients treated with INFα. Su et al. reported that patients presenting with virological response had lower circulating Treg [39]. Although peripheral blood changes may not necessarily reflect the marrow microenvironment [40], the easy accessibility of peripheral blood permits convenient monitoring of patients undergoing PegINFα therapy. Our data indicate that Treg play an important role in the pathophysiology of PV and ET and that failure of PegINFα to decrease circulating Treg predicts a poor molecular response.

## Conclusion

These results suggest that activation of the immune system by PegINFα can induce either a protective immune response that contributes to elimination of the malignant clone or a suppressive immune response, suggesting that increases in Treg prevent clearance of the mutated clone by effector CD8^+^ T cells. Further studies analyzing changes in immune cells in bone marrow may elucidate the role of PegINFα in inducing anti-tumor responses. We found that changes in peripheral blood T cell populations correlate with achievement of a molecular response to PegINFα.

## Additional files

**Additional file 1: FigS1.** Flow cytometry gating strategy. PBMC were processed as described in Material and Methods. Single cell suspensions were analyzed by flow cytometry. FSC and SSC were used to determine lymphocyte population. CD4^+^ T cells were analyzed for expression of CD25 and Foxp3 (Treg) (Panel B and F). In Panel C-D and G-H, CD3^+^ CD4^+^ CD25^+^ Foxp3^+^ Treg were analyzed using CD39 and HLA-DR (Panel C and G) and Helios and CD45RO (panel D and H). CD39, HLA-DR and Helios were used to characterize highly suppressive Treg. Treg were also assessed for proliferation by using Ki-67. Panels A-D represent analysis prior to PegINFα, and panels E-H after at least 10 weeks of PegINFα treatment.

**Additional file 2: FigS2.** Absolute numbers of Treg and highly suppressive Treg in peripheral blood of PV and ET patients are not significantly affected by Peginfα. PBMC were collected from patients with PV or ET treated with PegINFα or HU for at least 70 days (range 70-616 days, median 112 days, for PegINFα and range 113-2422 days, median 175 days, for HU treated patients). Lymphocytes were analyzed by flow cytometry using surface markers CD3, CD4, CD25, CD39, HLA-DR and intracellular markers Foxp3, Ki-67 and Helios. Panel A represents absolute numbers of CD4^+^ CD25^+^ Foxp3^+^ Treg cells. In panel B, the absolute number of Treg was analyzed at different time points after initiation of PegINFα treatment. Panels C-D-E show the absolute number of Ki-67^+^ Treg, Helios^+^ Treg, and CD39^+^/HLA-DR^+^ Treg.

**Additional file 3: FigS3.** Changes in numbers of Treg subsets in Peginfa treated patients based on molecular response. Patients treated with PegINFα were separated into R and NR subsets based on a 20% decrease in *JAK2* ^V617^ allelic burden. PBMC were analyzed as described above and fold changes compare to baseline were examined. Panel A represents 23 patient samples (10R and 13NR), panels B-C include 16 patients (6 R and 10 NR), and panel D includes 13 patients (4R and 9NR). A-C show the number of Treg, of dividing Treg, of CD39^+^ HLA-DR^+^ Treg and Helios^+^ Treg. Panel D shows CD38^+^ HLA-DR^+^ T cells in R vs NR.

## Abbreviations

PV: polycythemia vera
ET: essential thrombocytemia
MPN: myeloproliferative neoplasms
INFα: interferon alpha
PegINFα: pegylated-interferon alpha
HU: hydroxyurea
PBL: peripheral blood lymphocytes
Treg: CD4^+^ Foxp3^+^ regulatory T cells
Teff: CD4^+^ effector T cells
PBMC: peripheral blood mononuclear cells
