# Supplementary figures and images for: Changes in peripheral blood lymphocytes in polycythemia vera and essential thrombocythemia patients treated with pegylated-interferon alpha and correlation with JAK2V617F allelic burden
Source: Exp Hematol Oncol. 2016 Sep 27;5:28. doi: 10.1186/s40164-016-0057-y (PMC5037882; doi:10.1186/s40164-016-0057-y)

Supplemental Figure 1

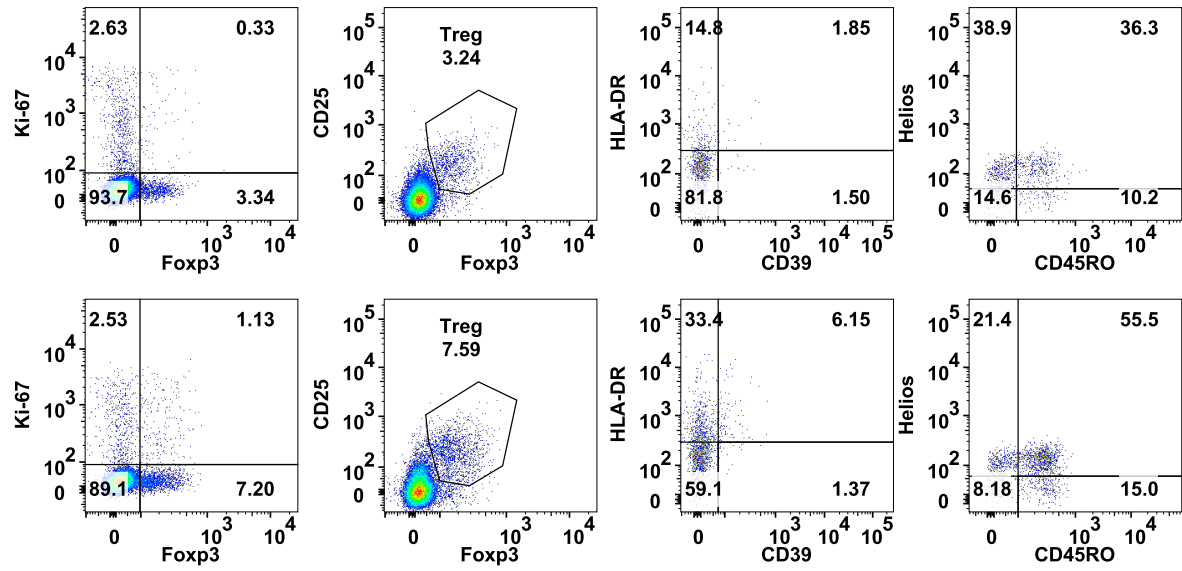

Supplement: Supplementary file 1 — Additional file 1: FigS1. Flow cytometry gating strategy. PBMC were processed as described in Material and Methods. Single cell suspensions were analyzed by flow cytometry. FSC and SSC were used to determine lymphocyte population. CD4+ T cells were analyzed for expression of CD25 and Foxp3 (Treg) (Panel B and F). In Panel C-D and G-H, CD3+ CD4+ CD25+ Foxp3+ Treg were analyzed using CD39 and HLA-DR (Panel C and G) and Helios and CD45RO (panel D and H). CD39, HLA-DR and Helios were used to characterize highly suppressive Treg. Treg were also assessed for proliferation by using Ki-67. Panels A-D represent analysis prior to PegINFα, and panels E-H after at least 10 weeks of PegINFα treatment. [file 40164_2016_57_MOESM1_ESM.pdf]

# Supplemental Figure 2

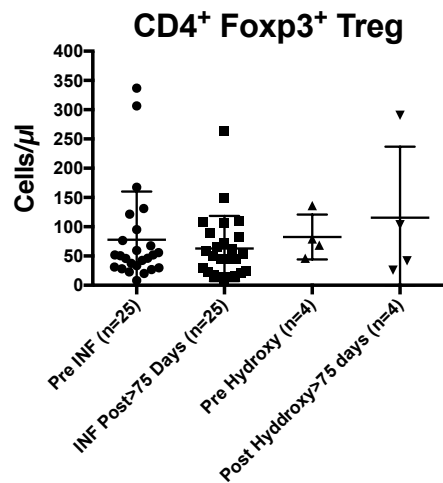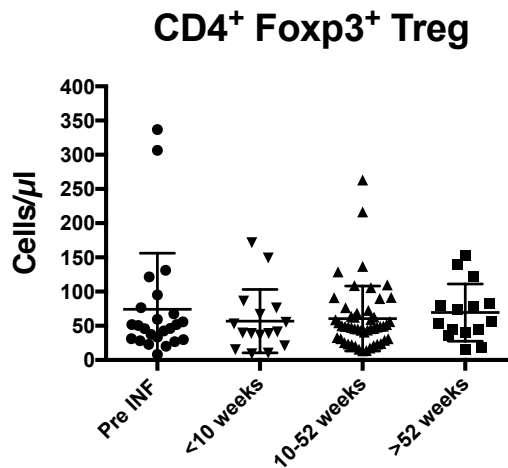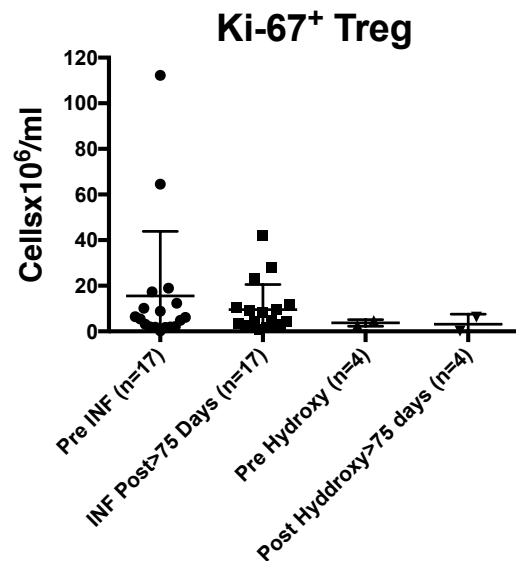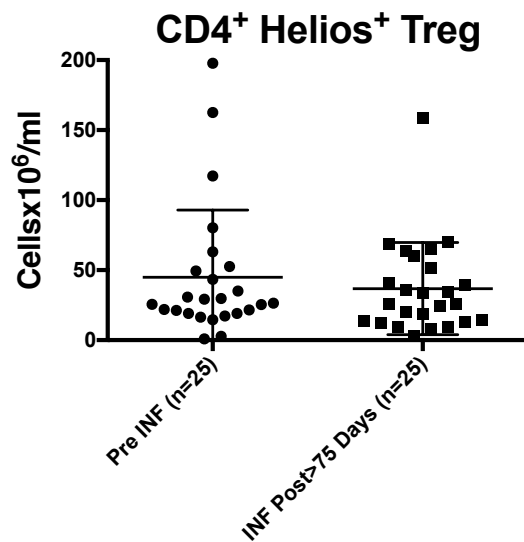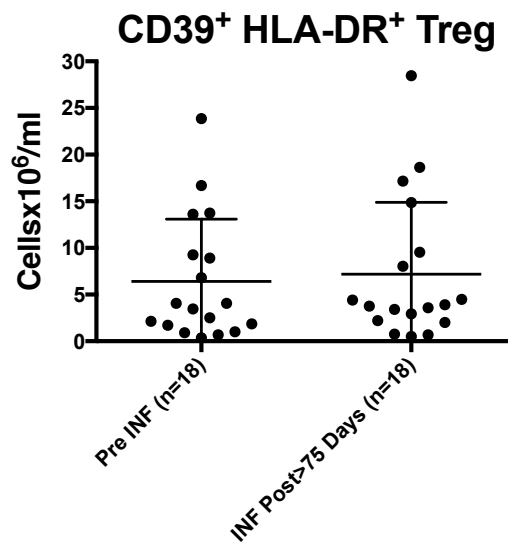

Supplement: Supplementary file 2 — Additional file 2: FigS2. Absolute numbers of Treg and highly suppressive Treg in peripheral blood of PV and ET patients are not significantly affected by Peginfα. PBMC were collected from patients with PV or ET treated with PegINFα or HU for at least 70 days (range 70-616 days, median 112 days, for PegINFα and range 113-2422 days, median 175 days, for HU treated patients). Lymphocytes were analyzed by flow cytometry using surface markers CD3, CD4, CD25, CD39, HLA-DR and intracellular markers Foxp3, Ki-67 and Helios. Panel A represents absolute numbers of CD4+ CD25+ Foxp3+ Treg cells. In panel B, the absolute number of Treg was analyzed at different time points after initiation of PegINFα treatment. Panels C-D-E show the absolute number of Ki-67+ Treg, Helios+ Treg, and CD39+/HLA-DR+ Treg. [file 40164_2016_57_MOESM2_ESM.pdf]
