# Supplementary material for: Changes in peripheral blood lymphocytes in polycythemia vera and essential thrombocythemia patients treated with pegylated-interferon alpha and correlation with JAK2V617F allelic burden
Source: Exp Hematol Oncol. 2016 Sep 27;5:28. doi: 10.1186/s40164-016-0057-y (PMC5037882; doi:10.1186/s40164-016-0057-y)

Supplemental Figure 3

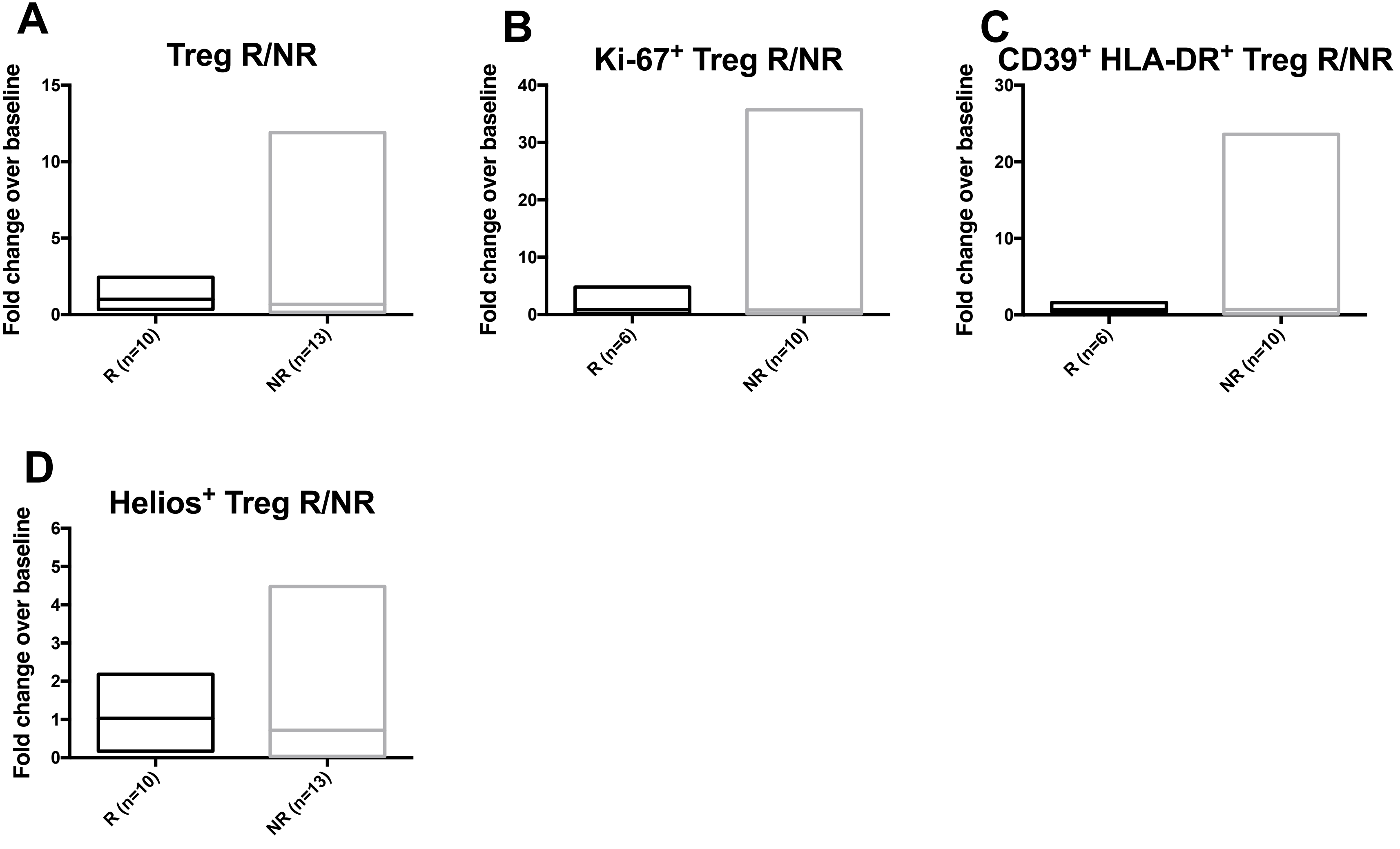

Supplement: Supplementary file 3 — Additional file 3: FigS3. Changes in numbers of Treg subsets in Peginfa treated patients based on molecular response. Patients treated with PegINFα were separated into R and NR subsets based on a 20% decrease in JAK2 V617 allelic burden. PBMC were analyzed as described above and fold changes compare to baseline were examined. Panel A represents 23 patient samples (10R and 13NR), panels B-C include 16 patients (6 R and 10 NR), and panel D includes 13 patients (4R and 9NR). A-C show the number of Treg, of dividing Treg, of CD39+ HLA-DR+ Treg and Helios+ Treg. Panel D shows CD38+ HLA-DR+ T cells in R vs NR. [file 40164_2016_57_MOESM3_ESM.pdf]
